# A Cross-Sectional Study on Healthcare Providers’ Perceived Compassion and Emotional Exhaustion Across Five Acute Geriatric Units: The Importance of Mutual Respect and Open Reflection

**DOI:** 10.3390/healthcare14121752

**Published:** 2026-06-17

**Authors:** Ruth Piers, Judith Hanssens, Jolien De Vos, Katrien Cobbaert, Inge Pattyn, Katrien Van Puyvelde, Barbara Vandervennet, Jille Gelders, Astrid Brys, Anja Velghe, Nele Van Den Noortgate, Shane Sinclair, Charlotte Boven

**Affiliations:** 1 Department of Geriatric Medicine, Ghent University Hospital, Corneel Heymanslaan 10, 9000 Ghent, Belgium; judith.hanssens@ugent.be (J.H.); jtodvos.devos@ugent.be (J.D.V.); astrid.brys@uzgent.be (A.B.); anja.velghe@uzgent.be (A.V.); nele.vandennoortgate@uzgent.be (N.V.D.N.); charlotte.boven@uzgent.be (C.B.); 2 Faculty of Medicine and Health Sciences, Ghent University, Corneel Heymanslaan 10, 9000 Ghent, Belgium; 3 Department of Geriatric Medicine, AZ Delta, Deltalaan 1, 8800 Roeselare, Belgium; katrien.cobbaert@azdelta.be; 4 Department of Geriatric Medicine, AZ Groeninge, President Kennedylaan 4, 8500 Kortrijk, Belgium; inge.pattyn@azgroeninge.be; 5 Department of Geriatric Medicine, AZORG, Merestraat 80, 9300 Aalst, Belgium; katrien.van.puyvelde@azorg.be; 6 Department of Geriatric Medicine, O.L.V. van Lourdes Ziekenhuis Waregem, Vijfseweg 150, 8790 Waregem, Belgium; barbara.vandervennet@ziekenhuiswaregem.be; 7 Department of Geriatric Medicine, AZ Oostende, Gouwelozestraat 100, 8400 Oostende, Belgium; jille.gelders@azoostende.be; 8 Compassion Research Lab, University of Calgary, Calgary, AB T2N 1N4, Canada; sinclair@ucalgary.ca; 9 Faculty of Nursing, University of Calgary, Calgary, AB T2N 1N4, Canada; 10 Cumming School of Medicine, University of Calgary, Calgary, AB T2N 1N4, Canada

**Keywords:** acute hospital, aged 80 and over, burnout, compassion, compassionate care, empathy, ethical decision-making, geriatrics, interprofessional collaboration, patient-centered care

## Abstract

**Highlights:**

**What are the main findings?**

- Levels of compassionate care differed across AGUs with a range in self-assessed mean team performance in compassion scores of 3.62 in the lowest scoring AGU and 4.28 in the highest scoring AGU, on a 5-point Likert scale.
- Team compassion scores were significantly related to the quality of the ethical climate, but not associated with individual healthcare provider emotional exhaustion.

**What are the implications of the main findings?**

- Although compassionate care has been historically seen as an attribute of individual healthcare providers, our findings suggest it may also be shaped by team dynamics.
- Mutual respect and open interprofessional reflection play key roles in achieving higher team compassionate care scores.

**Abstract:**

**Background/Objectives**: Despite growing evidence on the importance of compassionate care, it receives little attention in geriatrics literature. The aim is to study the variation and key components of team compassionate care and its relation to individual healthcare provider (HCP) emotional exhaustion in acute geriatric units (AGUs). **Methods**: A cross-sectional survey study, from February to April 2025, with a convenience sample of HCPs in five Belgian AGUs (70% response rate). Validated questionnaires were used: The Sinclair Compassion Questionnaire (SCQ), Emotional Exhaustion (EE) subscale of Maslach Burnout Inventory and Ethical Decision-Making Climate Questionnaire (EDMCQ). **Results**: In total, 118 HCPs participated: 11% team leaders, 28% paramedics, 61% nursing professionals. Mean AGU SCQ scores ranged from 3.62 to 4.28 on a scale from one (lowest) to five (highest). Multivariate linear regression models showed significant differences in team compassion scores across AGUs (estimate 0.084, *p* = 0.003) and increased with higher ethical climate scores (estimate per point on the EDMCQ 0.035, *p* < 0.001). Two EDMCQ domains (open interprofessional reflection and mutual respect) were associated with team compassionate care beyond the effect of AGUs, whereas demographics and self-reported emotional exhaustion were not (R^2^ = 0.283). Emotional exhaustion was significantly associated with professional role (estimate −3.004, *p* = 0.011), but not with AGUs (estimate 0.768, *p* = 0.269), ethical climate (estimate per point on the EDMCQ −0.248, *p* = 0.117) and team-based compassion scores (R^2^ = 0.115). Nursing professionals were significantly at higher risk for emotional exhaustion compared to paramedics (estimate 4.497, *p* = 0.037). **Conclusions**: The level of team compassionate care differed across AGUs and was correlated to the perceived ethical climate of the workplace, and not to individual HCP demographic variables or emotional exhaustion. Mutual respect and open interprofessional reflection may be specific areas for future research in improving high-quality compassionate care.

## 1. Introduction

Healthcare is increasingly shaped by technological advancements and efficiency-driven models. While these developments have enhanced diagnostic and therapeutic capacities, emerging evidence shows that the human side of medicine is decreasing [1,2]. A strong focus on technical performance risks overshadowing one of healthcare’s core purposes, namely the human experience of care [3]. This is especially relevant in Acute Geriatric Units (AGUs), where older patients often present with complex, multidimensional needs. These include increased vulnerability due to acute illness, care dependency, reduced decision-making capacity, end-of-life situations, behavioral problems, and complex family dynamics [4,5], all of which require a relational and individualized approach. These characteristics make AGUs a particularly relevant setting to study compassionate care in order to realize person-centered care.

Compassion can be defined as “a virtuous response that seeks to address the suffering and needs of a person through relational understanding and action” [6]. Encountering another person’s vulnerability triggers authentic responses aimed at comforting, alleviating, and preventing suffering [6,7,8]. Importantly, compassionate care is associated with improved patient outcomes, including reduced symptom severity, enhanced psychological well-being, and relief of suffering [9,10,11]. However, previous research in non-geriatric populations shows considerable variation in experiences of compassionate care, which is often reported as insufficient or lacking [12,13,14,15]. System pressures, such as high workloads, target-driven organizational cultures and HCP emotional exhaustion (EE), limit the capacity of HCPs to engage compassionately [16], resulting in a significant proportion of opportunities for compassionate interactions that are being missed [17,18].

Recent evidence shows a decline in work-related HCP well-being, a trend that has been further intensified by the COVID-19 pandemic [19,20]. The costs associated with diminished professional quality of life act as a serious burden to health systems worldwide [21,22,23]. In the care of older adults, attracting and retaining nurses and physicians has become a major challenge, not only in Belgium but across many Western countries [1,4,19,20,24,25]. Job attrition is closely linked to intention to leave and the ethical climate, which are shared norms on appropriate behavior and how to handle ethical issues, within organizations [26,27]. Creating care environments in AGUs that foster compassion may not only improve patient outcomes, but may also reduce staff turnover and burnout [1,24,25,28]. Burnout results from chronic workplace stress and encompasses EE, depersonalization, and a reduced sense of personal accomplishment [29]. EE is a key construct in healthcare-related burnout [29,30], and is most directly related to the emotional demands of patient care [31,32]. While compassionate care can enhance resilience among HCPs [16,28,33], it is also emotionally demanding, potentially resulting in increased emotional exhaustion, [31,32]. To cope, professionals may resort to emotional distancing as a form of self-protection against emotional exhaustion [34]. Recent literature therefore advocates integrating compassion training for individual HCPs within broader workplace interventions that promote emotional support and team-based approaches to managing challenging patient situations, which form the ethical climate [18,26,33,35,36,37]. The relationship between team compassionate care, ethical climate, and HCP EE is therefore deeply interconnected.

Despite scholars recognizing the importance of compassion in geriatric care [38,39,40,41], there remains a lack of evidence specifically focusing on older patients [42,43,44]. Limited insight exists into the variability of compassionate care across AGUs and the factors that explain this variation. Recent studies in geriatric care have primarily been qualitative in nature [40,41], limiting opportunities for statistical generalization.

## 2. Materials and Methods

### 2.1. Aim

The focus of this exploratory study was on compassionate care at the team level, its relation to HCP emotional exhaustion and on actionable strategies for implementation at the unit level. The research questions are as follows:

(a) What are the levels of team performance in compassionate care across five Belgian AGUs? Are there significant differences between AGUs?
(b) Is there an association between team performance in compassionate care and individual emotional exhaustion among HCPs in AGUs?
(c) Which factors, next to ethical climate and AGU site, are associated with team compassionate care? Which factors, next to ethical climate and AGU site, are associated with individual HCP well-being?

We hypothesized that there would be variation in the team compassionate care scores across AGUs, and that a higher perceived team compassion would be linked to higher individual HCP emotional exhaustion and higher ethical climate scores.

### 2.2. Study Design and Setting

This multicenter observational study was conducted via a cross-sectional survey with HCPs working in five Belgian AGUs. Eligible AGUs included general geriatric, orthogeriatric or mixed wards. Psychogeriatric units were not eligible. The five participating AGUs were located in non-university hospitals, with one ward being an orthogeriatric ward and the other four being mixed wards. Across the five participating AGUs, the mean number of beds was 26.6 (range 24–30), the mean patient-to-nurse ratio across shifts was 2.7 (range 2.2–3.0), and the mean total team size was 33.4 HCPs (range 26–40), suggesting relatively similar organizational conditions across sites. In Belgium, there is also a legal framework that defines the parameters for staffing, financing, and eligible patient population for AGUs, thereby ensuring consistency across units and enhancing their comparability in research studies to reduce bias. Moreover, patient populations and organizational structures in AGUs are largely comparable worldwide [45,46].

From February to April 2025, five local investigators recruited HCPs from each of the AGUs. Recruitment was conducted by informing eligible team members about the study during scheduled staff meetings. After this initial information session, all eligible team members received the survey link by e-mail, and two reminder e-mails were sent to maximize participation.

### 2.3. Study Population

HCPs from each of the five interprofessional AGU teams were invited to participate. Team members included nursing professionals (registered nurses and licensed practical nurses, nursing aides), paramedics (physical, speech and occupational therapists, psychologists, social workers, dieticians, spiritual care providers), other team members (logistic support, administrative staff) and interprofessional team leaders of the AGU (doctors and head nurses). As this study was exploratory in nature, no formal a priori statistical power calculation was performed. Instead, sample size considerations were guided by commonly used recommendations for multivariable regression modelling, including the traditional rule of thumb suggesting approximately 10 observations per predictor variable in the full model [47]. To reduce bias and ensure rigor, a minimum response rate of 60% per team was required. In order to obtain the maximum response rate, two e-mails containing the survey link were sent to all eligible team members.

### 2.4. Outcomes and Instruments

The primary outcomes were self-reported team-based compassionate care measured by the Sinclair Compassion Questionnaire-Short Form-Healthcare Provider Team Assessment (SCQ-SF-HCPTA) and individual HCP self-reported emotional exhaustion by the Emotional Exhaustion (EE) subscale of the Maslach Burnout Inventory. The ethical climate, measured by the Ethical Decision-Making Climate Questionnaire (EDMCQ), demographics, and the AGU site, was used to identify factors associated with both scores.

The SCQ is a validated instrument for quantifying compassion in healthcare from multiple perspectives (e.g., patients, family, HCP). It studies outcomes valued by the patient, such as ‘feeling heard and understood by their HCP’ and ‘being valued as a person’ [48,49]. The Belgian version of the 5-item SCQ-SF-HCPTA was used, with each item rating on a five-point Likert scale ranging from 1 (strongly disagree) to 5 (strongly agree). Prior to its use in this study, the questionnaire underwent a formal translation and cross-cultural adaptation process. This included forward translation, back translation, and expert review, followed by cognitive interviews with 20 AGU patients to assess the comprehensibility and cultural appropriateness of compassion-related wording. Subsequently, an additional expert review and a final consensus meeting were conducted to adapt the team-assessment version for HCPs. HCPs were asked to rate the team-based compassionate care delivered, using statements such as ‘In my unit, patients are seen as a person and not just as a patient’. The total score is calculated as the mean of the five items, resulting in a theoretical range from 1 to 5. A higher SCQ score is indicative of higher team-based compassionate care. Given that the SCQ-SF-HCPTA was only recently developed, we conducted an exploratory factor analysis using minimum residual estimation to examine the underlying factor structure of the SCQ items. The Kaiser–Meyer–Olkin measure indicated good sampling adequacy (KMO = 0.80), with item-level values ranging from 0.77 to 0.86. Bartlett’s test of sphericity was significant (χ^2^(10) = 253.60, *p* < 0.001), indicating that correlations between items were good enough for factor analysis. Factor loadings ranged from 0.61 to 0.83, explaining 53% of the total variance. As a single factor was extracted, no rotation was applied. Internal consistency was high (Cronbach’s alpha = 0.85). The measure was positively correlated with the HCP-reported Hospital Consumer Assessment of Healthcare Providers and Systems instrument [50] (r = 0.67, *p* < 0.001), in which HCPs rate their hospitals on a scale from 0 to 10 (best). The SCQ, user manual, and other resources are freely available to individuals and researchers at www.compassionmeasure.com.

EE is assessed by a 9-item subscale of the well-validated Maslach Burnout Inventory. Each item is rated on a seven-point Likert scale from 0 (never) to 6 (every day), indicating how frequently respondents experience the described feeling associated with burnout. The total EE score is calculated by summing the responses to all 9 items, resulting in a theoretical range of 0 to 54. Higher EE scores indicate lower well-being among healthcare Individuals. Persons with a score of 27 or higher are considered to have symptoms of burnout. Since intention to leave one’s job is also a marker of work-related well-being [31], we assessed team members’ desire to leave their job by asking them to respond to the statement ‘I am thinking of leaving my job’ (‘agree’ or ‘not agree’).

We selected the EDMCQ because it captures dimensions of ethical climate that are highly relevant in acute geriatric care, including interprofessional reflection, mutual respect, leadership, ethical awareness, and nurses’ involvement in end-of-life decision-making. These aspects are particularly pertinent in AGUs, where teams care for frail older adults with multimorbidity, cognitive vulnerability, and complex end-of-life decisions. The ethical climate was assessed by the Ethical Decision-Making Climate Questionnaire (EDMCQ), a 30-item validated questionnaire that consists of seven domains: (1) “self-reflective and empowering leadership of team leaders”, (2) “open and interdisciplinary reflection”, (3) “not avoiding end-of-life decisions”, (4) “mutual respect within the interdisciplinary team”, (5) “active involvement of nurses in end-of-life care and decision-making”, (6) “active decision-making by team leaders” and (7) “ethical awareness” [26,51]. All 30 items are rated on either a 4-point or 5-point Likert scale ranging from 1 (strongly disagree) to 4 or 5 (strongly agree). The total EDMCQ score is calculated as the sum of all item scores, theoretically ranging between 30 and 142. The higher the EDMCQ score, the better the ethical climate. More detailed information on the theoretical framework and instruments can be found elsewhere [26].

The HCP survey comprised two parts: (1) demographics and personal characteristics (including EE), and (2) ethical climate and care in the department (including the EDMCQ and the SCQ). Participants who did not complete part 1 were excluded from the analysis. Respondents with missing data in part 2 were retained, and analyses were conducted using available data. The number of observations may therefore vary slightly across analyses. For example, for the regression analysis on SCQ, 102 complete cases were used, with 16 cases with data missing completely at random. Because the proportion of missing data was low and missing at random, sensitivity analysis was not performed.

To protect anonymity of the individual HCPs and the five AGUs, only overall percentages per AGU are reported, and the AGUs are not identified by name.

### 2.5. Statistics

We used IBM SPSS Statistics software version 28 (IBM Corp., Armonk, NY, USA).

- Descriptive statistics

Categorical data were summarized using frequencies and percentages, while continuous data were reported as means and standard deviations (SD). Due to rounding, reported proportions may not sum precisely to 100%.

B. Group comparisons

To compare variables between AGUs and professional roles, a Student’s *t*-test or one-way ANOVA (Table 1) were used for continuous variables, and Pearson chi-square for categorical data (Table 2).

C. Correlation analyses

To test a univariate linear relationship between team performance in compassionate care (SCQ-SF-HCPTA) and EE as reported by the individual HCP, a Pearson correlation coefficient was calculated. We also tested univariate correlation between team mean scores of SCQ-SF-HCPTA and EE with the non-parametric Spearman’s rho test.

D. Multivariable analyses

For both primary outcomes, SCQ-SF-HCPTA and EE, multivariate linear regression models were conducted to identify associated factors. Given the sample size, the number of predictors included in the multivariate models was limited, and forward selection was applied to reduce the risk of overparameterization. Each model included AGU site, the total EDMCQ score, and relevant HCP characteristics as independent variables. First, AGU and EDMCQ were entered as fixed effects in the linear regression model to address the primary research questions: whether significant differences exist between AGUs’ self-assessed team-based compassionate care and whether the ethical climate is associated with this outcome. Second, age, gender, professional role, work hours, and EE (or vice versa, the SCQ-SF-HCPTA score in the model with outcome EE) were considered in forward selection because we also wished to examine whether the outcomes were associated with individual HCP characteristics beyond AGU and ethical climate. Separate subgroup analyses by profession were not prespecified, because it was not our main interest and because the limited sample size of several professional subgroups would not have supported robust profession-specific comparisons. The total EDMCQ score was analyzed first as an overall quantitative measure of the ethical climate (Table 3 and Table 4, model A). Subsequently, a second model (Table 3 and Table 4, model B) was fitted in which the seven individual EDMCQ factor scores replaced the total EDMCQ score, because these factor scores provide additional content-wise information on specific dimensions of the ethical climate and may therefore yield more interpretable and actionable findings [52,53]. For this model, we implemented similar entry and removal criteria and also selected the enter and forward stepwise selection.

E. Assumption checking and reporting

For all linear models, assumptions of linearity, independence, homoscedasticity, normality of residuals, and absence of multicollinearity were assessed. Normality of residuals was verified graphically using Q–Q plots. Model performance was evaluated based on the proportion of explained variance (*R*^2^). The exact *p*-values are reported, with statistical significance defined as *p* < 0.05.

## 3. Results

A total of 118 eligible participants were recruited from five different AGUs, attaining an overall response rate of 70% (118/167). Reasons for non-participation and characteristics of non-responders were not sought, to uphold the voluntary nature of participation. Therefore, it was not possible to report on the nonresponse bias. Table 1 summarizes the overall distribution of HCP characteristics, reporting both the overall sample scores and those stratified by AGU.

The study sample comprised 11% interprofessional team leaders (head nurses and doctors), 28% paramedics, and 61% nursing professionals. Two-thirds of the participants were employed at 80% full-time equivalent or higher.

In this study sample, 10% of HCPs (12/116) reported the intention to leave their job (agreeing on ‘I am thinking of leaving my job’). This included 14% of nursing professionals, 0% of interprofessional team leaders and 6% of paramedics or others (*p* = 0.207). Intention to leave was significantly associated with EE, with mean EE scores of 12.18 among HCP providers without intention to leave compared to 27.83 among those with such an intention (*p* < 0.001).

### 3.1. Ethical Climate (EDMCQ)

**Figure A1:**
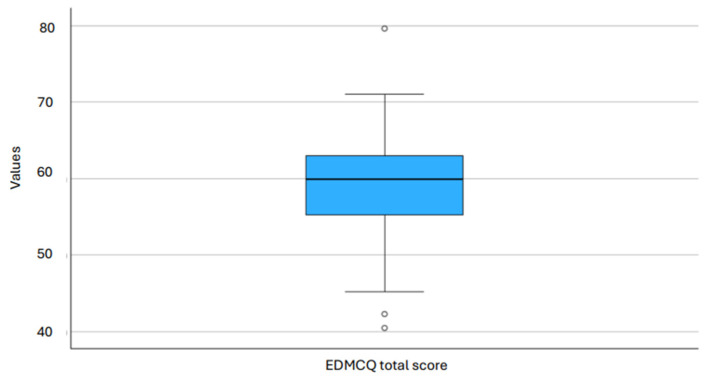
Total score of the EDMCQ: A 30-item questionnaire with total scores ranging theoretically from 30 (low) to 142 (high).

The mean total EDMCQ score was 59.38, with observed scores ranging from 40.49 to 79.61 (Table 1). More detailed results can be found in Table A1 and Figure A1 in Appendix A. Table 2 and Table A2 (Appendix A) provide insight into the level of agreement on individual items of the EDMCQ. Results indicate that HCPs agreed on less than 60% of the items concerning feeling safe to speak up, the availability for interprofessional reflection on patient cases, and the vulnerability of team leaders. Almost all HCPs agreed on the items in the factors ‘culture of not avoiding end-of-life decisions’ and ‘ethical awareness’ (Table A2).

### 3.2. Levels of Team Compassionate Care (SCQ) and Individual HCP Emotional Exhaustion (EE) and Their Univariate Linear Relation

**Figure A2:**
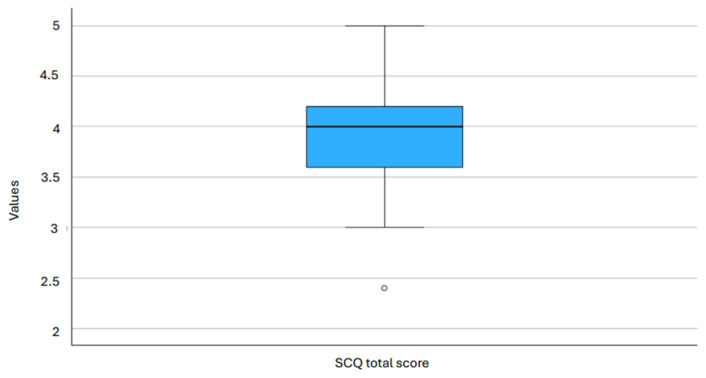
Total score of the SCQ: A five-item questionnaire with total scores ranging theoretically from 1 (low) to 5 (high).

**Figure A3:**
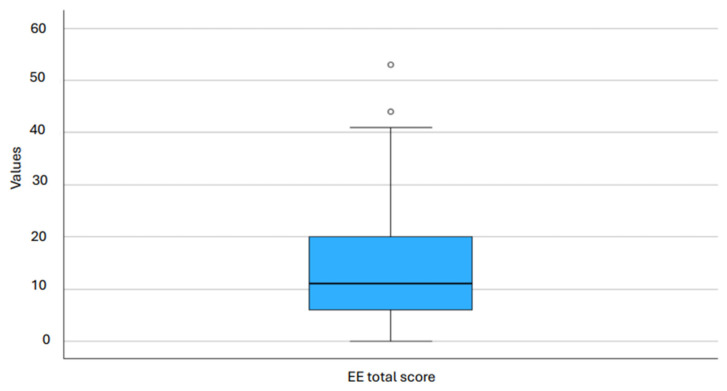
Total score of the EE: A nine-item questionnaire with total scores ranging theoretically from 0 (low) to 54 (high). Scores of 27 or higher are considered to have symptoms of burnout.

The mean SCQ-SF-HCPTA score was 3.98, with a minimum score of 2.40 and a maximum of 5.00 (see Table A3 and Figure A2 in Appendix B). When comparing AGUs, mean team-based compassionate care scores ranged from 3.62 to 4.28 (Table 1) on a scale from 1 to 5, with a difference of 0.33 found to be clinically significant [49]. The mean EE score across all AGUs was 13.91, with more detailed results provided in Table A4 and Figure A3 (Appendix C).

**Figure A4:**
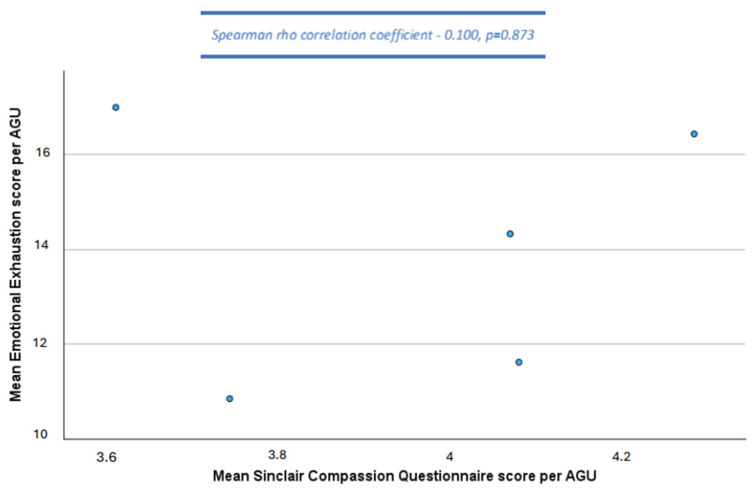
Relationship between SCQ and EE. This is a visual representation of the linear relationship between SCQ and EE. The dots represent mean observed scores for the SCQ and EE for each of the five AGUs.

No linear relationship was observed between individual HCPs’ perception of team compassion and his/her individual EE score, with a Pearson correlation coefficient of −0.088 (95% CI: −0.277 to 0.107). On the team level, there was no correlation between the mean compassion score and mean EE score within each AGU (Spearman rho correlation coefficient—0.100, *p* = 0.873) (Table 1 and Figure A4 in Appendix D).

### 3.3. Factors Associated with Team Compassionate Care (SCQ) and Individual HCP Emotional Exhaustion (EE) in Multivariate Analyses

As shown in model A of Table 3, self-assessed team compassionate care scores, as reflected in the SCQ-SF-HCPTA total score, differed significantly across AGUs (estimate = 0.084, *p* = 0.003), while EE scores did not (estimate = 0.768, *p* = 0.269) in the multivariate analysis (model A of Table 4).

**Figure 1:**
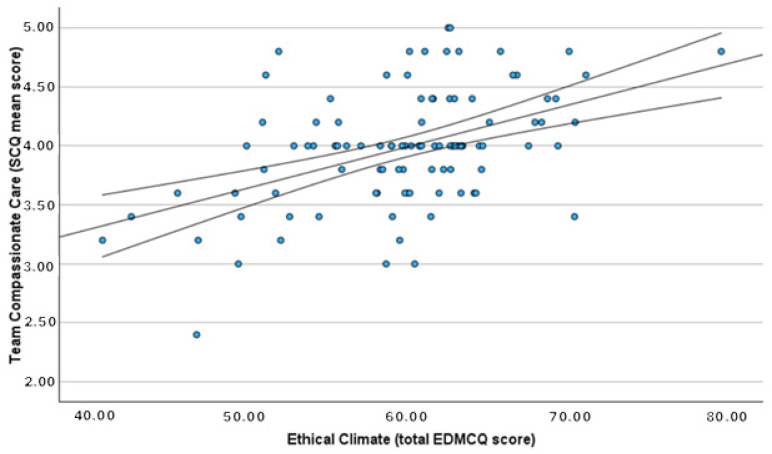
Team performance in Compassionate Care as a function of the Ethical Climate. This is a visual representation of the linear relationship between SCQ and EDMCQ, with the 95% CI around the regression line based on the linear regression model as presented in model A of Table 3. The dots represent the observed values. Abbreviations: EDMCQ = Ethical Decision-Making Climate Questionnaire; SCQ = Sinclair Compassion Questionnaire.

Model A of Table 3 and Figure 1 illustrate that the SCQ-SF-HCPTA total score increased significantly with higher ethical climate (estimate per point on the EDMCQ total score = 0.035, *p* < 0.001). 

R^2^ of this multivariate model was 0.283. Model B of Table 3 shows that two domains of the EDMCQ (factor 2 ‘practice and culture of open interdisciplinary reflection’ and factor 4 ‘culture of mutual respect within the interdisciplinary team’) were significantly associated with higher team compassionate care scores, beyond the effect of AGU. Professional role was marginally significant (*p* = 0.050), therefore rejected from the stepwise regression model. All other HCP variables (including EE) were not significantly associated.

Model B of Table 3 shows that two domains of the EDMCQ (factor 2 ‘practice and culture of open interdisciplinary reflection’ and factor 4 ‘culture of mutual respect within the interdisciplinary team’) were significantly associated with higher team compassionate care scores, beyond the effect of AGU. All other HCP variables (including EE) were not significantly associated, except for professional role, which was marginally significant (*p* = 0.054).

Emotional exhaustion was significantly associated with professional role (estimate = −3.004, *p* = 0.011), but not with AGU (estimate = 0.768, *p* = 0.269) and not with ethical climate (EDMCQ) (estimate per point on the EDMCQ total score = −0.248, *p* = 0.117) and not with compassionate care scores (R^2^ of this multivariate model was 0.115, model A of Table 4). Nursing professionals were significantly at higher risk for EE compared to paramedics (estimate = 4.497, *p* = 0.037). In the model with the seven EDMCQ domains, the higher score for factor 4 ‘culture of mutual respect within the interdisciplinary team’ was associated with less EE (model B of Table 4), aside from professional role.

## 4. Discussion

Our study presents novel findings on compassion in acute geriatric care and is, to the best of the authors’ knowledge, the first time that HCPs’ perspectives on team-based compassion have been measured [54]. By comparing compassion scores across similar units of care, we were able to reveal insights into the variation of compassionate care and how the team context influences this. The proportion of explained variance in the regression models was modest, indicating that relevant determinants of compassionate care and emotional exhaustion were not fully captured in the present study. Potentially important unmeasured factors may include organizational culture, workload, leadership style, and individual psychological characteristics. Nonetheless, our findings provide actionable directions for both research and practice, in Belgium and internationally. Although AGUs are referred to by various terms, such as Acute Frailty units, Acute Care for Elders units, or Comprehensive Geriatric Assessment units, their patient populations and organizational structures are comparable [45,46].

### 4.1. Variation in Team Compassion Scores Across AGUs

Although compassion is often viewed as an attribute of individual HCPs [39], this study assessed compassion at a team level. The study reveals that levels of self-assessed team-based compassionate care varied across AGUs and that compassion scores were related to the ethical climate of each unit, and not to individual HCP factors. A compassionate care climate seems to be a key factor that affects how well HCPs can provide compassionate care to older patients in the hospital. This is especially important in AGUs because staff work in different shifts and care for patients who are often vulnerable and cannot speak up for themselves. These patients depend heavily on others, in every shift, to receive kind and compassionate care [4,35,38,55,56].

Sinclair et al. [49] found that ratings of compassionate care were lower in long-term care facilities than in acute care (average scores of 3.97 versus 4.39). We found similar variance in our study comparing AGUs, with mean SCQ-SF-HCPTA scores ranging from 3.62 to 4.28 on a scale from 1 to 5, with a difference of 0.33 considered to be clinically significant [49]. This suggests that while team compassion in AGUs is satisfactory, the degree of compassionate care varies considerably depending on the specific AGU team [39,57].

### 4.2. Association Between Team Compassion and Individual HCP Emotional Exhaustion

Contrary to our expectations, we did not find a link between team-based compassionate care (“how well is my team doing?”) and HCP EE. Previous research that looked at the individual HCP level found that a person’s own ability to provide compassionate care (“how well am I doing?”) was linked to burnout [33,58,59]. Other research highlights that compassionate care cannot rely only on individual efforts [7,38,60,61], but must also be supported at the organizational and system level to be sustained. For example, HCPs working in environments that do not match their values report both lower ability to provide compassion and higher burnout [62], which can be understood as moral distress. Moral distress arises when external constraints prevent acting according to one’s values [36]. Our findings seem to suggest that HCPs’ emotional exhaustion is mainly influenced by their own ability to deliver compassionate care, rather than by the team’s overall performance. This supports the view of burnout as a problem rooted in the individual care relationship. This has important implications for practice: while managers may focus on improving team performance, HCPs are more likely to engage in interventions that give them the space to provide compassionate care. This is especially the case for nurses who are at highest risk of EE in AGUs [5]. Nursing professionals may need additional support from management and team members to cope with feelings of powerlessness, influence team practices, and improve care for all patients in the AGU, moving from an individual to a more team-based perspective [5,33,35,37]. Overall, these findings underline that, alongside training in compassion, workplace conditions and organizational support are essential to sustain compassionate care [18,26,33,35,36,37].

Another explanation for not finding an association between individual EE and team compassionate care scores is that other factors may be involved, as suggested by the relatively low R^2^ of the multivariate model. In this study, we deliberately focused on actionable variables relevant for practice, while previous research shows that burnout, of which emotional exhaustion is a core component, is also influenced by individual factors such as anxiety, personality traits, and social functioning [63]. Well-being in general is multidimensional; it might be that other dimensions, such as resilience, job satisfaction or a sense of coherence have played a role in the previously reported studies. Additionally, other moderating or mediating factors may play a role in increasing HCP burden, including death and discharge rates, the presence of volunteers, and organizational factors such as staffing and bed turnover [63,64].

Future research with larger samples should explore how mediating or moderating factors may influence the relationship between AGU and compassionate care. In our study, other HCP characteristics were not significant, while professional role was only marginally significant (*p* = 0.050), possibly due to limited statistical power.

### 4.3. Association Between Ethical Climate and Team Compassion

Importantly, this study provides new evidence that the ethical climate, and more in particular, a culture of mutual respect and shared reflection, plays a key role in compassionate care in the complex setting of AGUs. Recent literature underscores the crucial role for team leaders (doctors and head nurses) in enhancing team functioning and open interprofessional reflection [5,57,65,66,67,68]. Feeling underappreciated and undervalued by their superiors was cited as a major impeder of compassionate care in Canadian long-term care facilities; HCPs in that study pointed out that the lack of compassion between professional groups often made it difficult or even hypocritical to provide compassion to patients [38]. This is in line with studies in industry or other specialties showing that high-performing teams are characterized by clear leadership, shared values and mutual respect for each other’s roles [37,69]. Future research would benefit from interventional designs to help establish causal relationships between team compassionate care, ethical climate and HCP emotional exhaustion.

### 4.4. Strengths and Limitations

The innovative character and the multicenter dataset with high response rate are the main strengths of this study. The main limitation of this exploratory study is the convenience sample of AGUs without a priori power analysis, limiting the statistical robustness of findings. Due to its exploratory nature and the relatively small sample size, results should be interpreted with caution, as our study may be underpowered to detect associations.

Selection bias may have occurred, as participation was voluntary, and those more interested in compassionate care, ethical climate, or work-related well-being may have been more likely to respond. Although we aimed to mitigate this risk by requiring a minimum response rate of 60% per team and obtained an overall response rate of 70%, systematic differences between responders and non-responders cannot be excluded.

Another limitation is the cross-sectional nature of the data, preventing causal interpretation of the key concepts. The observed associations do not allow for establishing a directional relationship between ethical climate, compassion and emotional exhaustion.

Forward stepwise selection was used to explore whether individual HCP characteristics were associated with compassionate care and emotional exhaustion beyond AGU and ethical climate. Although this approach can be useful for exploratory screening, it has recognized limitations. The resulting models should therefore be interpreted as exploratory and validated in future studies using larger samples and theory-driven modelling strategies.

This study predominantly focuses on the HCP perspective; however, compassionate care is fundamentally patient-centered. Nonetheless, studies have shown that team members are very capable of estimating the quality of the care on their wards [5,31]. This study shows that EE was not related to compassion scores, supporting the objectivity of the SCQ as appraised by HCPs. It would still be valuable for future research to incorporate the perspectives of patients and their families to better understand compassionate care in AGUs.

### 4.5. Future Research

Understanding the factors that shape compassionate care is key to enhancing care quality and HCPs’ emotional well-being. In our cross-sectional survey, the observed associations do not allow for establishing a directional relationship between ethical climate, compassionate care and emotional exhaustion.

In contrast to previous literature, we did not find a link between compassionate care and individual HCPEE: qualitative research methodology would help to deepen the understanding of the relationship between compassionate care for patients and for HCPs.

Future studies with larger sample sizes should further investigate potential effect modification by profession and other mediating and moderating variables in the relationship between AGU and the outcome.

Comparisons between workplaces in different countries would add to the state of the art in this field of humanistic medicine.

In order to identify factors that help in improving compassionate care, intervention studies in the complex setting of AGUs are warranted.

### 4.6. Implications for Practice

The study results underline the need for investment in open interprofessional reflection in AGUs in order to improve team-based compassionate care. There are several promising interprofessional interventions available, such as Schwartz Rounds [70], exposure by dialogue [71], group ethical reflection [72,73], and compassion training programs [74]. However, most of these interventions have been evaluated at the hospital-wide level and have not been specifically studied within acute geriatric units (AGUs), except for the exposure by dialogue. Given the unique characteristics of AGUs, such as complex patient needs, cognitive vulnerability, and ethical challenges, there is a need to assess whether these interventions require adaptation for this setting specifically.

Our results also support the view that special attention is needed for nursing professionals who need the support of their leaders to speak up; otherwise, these interventions may lead to increased EE [37,57,71,72].

## 5. Conclusions

Our cross-sectional survey shows that the level of team compassion in AGUs is satisfactory; however the degree of compassionate care differs considerably between AGUs. To improve team compassionate care, it may be pivotal to work on mutual respect and shared reflection within the interprofessional team. Contrary to what we hypothesized, no association was found between team-based compassionate care scores and individual HCP emotional exhaustion.

## Figures and Tables

**Table 1:** HCP Characteristics, Ethical Climate, Team-based Compassionate Care and HCP Emotional Exhaustion reported for each AGU site.

	Total Sample	AGU a	AGU b	AGU c	AGU d	AGU e	*p*-Value
Response rate	118/167 (70.1%)	80.8%	62.1%	73.0%	71.4%	67.5%	
Gender							0.254
Male	15/116 (12.9%)	10%	16.7%	25.9%	8%	3.8%	
Age category							0.612
Less than 35 years	47/118 (39.8%)	38.1%	22.2%	37%	48%	48.1%	
35–44 years	32/118 (27.1%)	33.3%	38.9%	33.3%	16%	18.5%	
More than 44 years	39/118 (33.1%)	28.6%	38.9%	29.6%	36%	33.3%	
Partner/life companion (yes)	105/117 (89.7%)	85.7%	100%	92.6%	83.3%	88.9%	0.440
Child/children (yes)	79/118 (66.9%)	66.7%	77.8%	66.7%	56%	70.4%	0.653
Role							**0.022**
Nursing professionals *	72/118 (61.0%)	71.4%	44.4%	70.4%	60%	55.6%	
Paramedics or other	33/118 (28.0%)	28.6%	27.8%	25.9%	24%	40.7%	
Interprofessional team leaders	13/118 (11.0%)	0%	27.8%	3.7%	16%	3.7%	
Work hours							0.065
Less than 80%	41/117 (35.0%)	57.1%	38.9%	30.8%	16%	37%	
80% or more	76/118 (65.0%)	42.9%	61.1%	69.2%	84%	63%	
Ethical Climate: total EDMCQ score (mean, SD)	59.38 (6.41)	61.61 (6.24)	60.89 (4.48)	59.49 (5.24)	57.70 (7.09)	58.18 (7.44)	0.211
Team-based Compassionate Care: SCQ score (mean, SD)	3.98 (0.48)	4.08 (0.31)	3.62 (0.33)	4.07 (0.41)	4.28 (0.50)	3.75 (0.52)	**<0.001**
HCP individual Emotional Exhaustion score (mean, SD)	13.91 (10.28)	11.62 (9.07)	17.00 (11.70)	14.33 (11.10)	16.44 (10.92)	10.85 (7.95)	0.157
HCP individual intent to leave job (% agree)	12/116 (10.3%)	4.8%	11.1%	7.7%	12.5%	14.8%	0.806

Legend. AGU a–e = refers to the different participating AGU sites (intentionally anonymized); HCP = Healthcare provider; EDMCQ = Ethical Decision-Making Climate Questionnaire, 30-item questionnaire with theoretical range between 30 and 142; SCQ = Sinclair Compassion Questionnaire, 5-item questionnaire with mean score theoretically ranging between 1 and 5; Emotional exhaustion is assessed by a 9-item subscale of the well-validated Maslach Burnout Inventory (MBI-EE), with theoretical range between 0 and 54. Bold formatting is used to illustrate significant values. * Among nursing professionals, there were 59 registered nurses and licensed practical nurses and 13 nursing aides; among paramedics and others, there were 3 physical therapists, 9 occupational therapists, 1 psychologist, 6 social workers, 4 speech therapists, 2 dieticians, 2 spiritual care providers/pastors, 4 logistic support staff, and 2 administrative staff; among team leaders, there were 8 doctors and 5 head nurses.

**Table 2:** Proportion of agreement for each of the individual items within Factor 2 and Factor 4 of the EDMCQ, along with the mean scores per factor reported for each AGU site.

	Total Sample (n = 108)	AGU a (n = 24)	AGU b (n = 25)	AGU c (n = 14)	AGU d (n = 21)	AGU e (n = 24)	*p*-Value
*Factor 2: Practice and culture of open interdisciplinary reflection* (mean score: 15.07; observed range: [7.75–21.05]) In my unit, there are regular opportunities for open informal dialogue between HCPs.	80 (74%)	15 (62.5%)	19 (76%)	13 (92.9%)	17 (80.9%)	16 (66.7%)	0.581
In my unit, there is regular structured and formal dialogue between the various disciplines within the team to discuss patient care.	83 (76.9%)	14 (58.4%)	18 (72%)	13 (92.9%)	17 (80.9%)	21 (87.5%)	0.530
In my unit, we regularly reflect on the quality of care provided from the various points of view of the staff.	54 (50%)	10 (41.7%)	12 (48%)	7 (50%)	11 (52.4%)	14 (58.4%)	0.464
In my unit, the teams are well coordinated/managed.	69 (63.9%)	20 (83.3%)	15 (60%)	13 (92.9%)	12 (57.1%)	9 (37.5%)	**0.037**
In my unit, there is an open and constructive culture in the department such that criticism can be easily expressed.	47 (43.5%)	10 (41.7%)	9 (36%)	9 (64.3%)	10 (47.6%)	9 (37.5%)	**0.010**
In my unit, discussions about patients lead to greater understanding and agreements.	61 (56.5%)	15 (62.5%)	13 (52%)	10 (71.4%)	11 (52.4%)	12 (50%)	0.989
The culture in my department makes it easy to learn from the errors of others.	70 (64.8%)	18 (75%)	15 (60%)	8 (44.4%)	15 (71.5%)	14 (58.4%)	0.728
In my unit, there is a structured, formal debriefing after difficult patient care situations.	67 (62.1%)	16 (66.7%)	13 (52%)	9 (64.3%)	15 (71.5%)	14 (58.4%)	0.674
*Factor 4: Culture of mutual respect within the interdisciplinary team* (mean score: 7.21; observed range: [3.96–8.90])							
In my unit, I am always regarded and addressed by everyone in the team as a full-fledged team member.	91 (84.3%)	19 (79.2%)	19 (76%)	14 (100%)	20 (95.3%)	19 (79.1%)	0.351
In my unit, team members from another discipline respect my work.	86 (79.7%)	14 (58.3%)	21 (84%)	12 (85.7%)	20 (95.3%)	19 (79.1%)	**0.039**
In my unit, I have confidence in the professional competence of my team members.	87 (80.5%)	18 (75%)	19 (76%)	10 (71.4%)	20 (95.3%)	20 (83.3%)	0.282

Legend. AGU a–e = refers to the different participating AGU sites (intentionally anonymized); HCP = Healthcare provider; EDMCQ = Ethical Decision-Making Climate Questionnaire, 30-item questionnaire with theoretical range between 30 and 142. Bold formatting is used to indicate significant values.

**Table 3:** Linear regression for Team-based Compassionate Care in Acute Geriatric Units.

Variables	Unstandardized B	Coefficients Std. Error	Standardized Coefficients Beta	t	*p*-Value	Collinearity Tolerance	Statistics VIF
MODEL A with EDMCQ total score							
Acute Geriatric Unit	0.084	0.028	0.257	3.013	**0.003**	1.000	1.000
Ethical Climate (total EDMCQ)	0.035	0.006	0.470	5.523	**<0.001**	1.000	1.000
Constant	1.645	0.390		4.221	**<0.001**		
MODEL B with the EDMCQ factor scores							
Acute Geriatric Unit	0.055	0.029	0.168	1.879	0.063	0.921	1.086
F2. Open interdisciplinary reflection	0.061	0.021	0.285	2.886	**0.005**	0.754	1.326
F4. Mutual respect within the interdisciplinary team	0.115	0.045	0.264	2.578	**0.011**	0.703	1.422
Constant	2.058	0.322		6.382	**<0.001**		

Legend. Abbreviations: EDMCQ = Ethical Decision-Making Climate Questionnaire, AGU = Acute Geriatric unit, SCQ = Sinclair Compassion Questionnaire; F = factor. Bold formatting is used to indicate significant values. MODEL A with total EDMCQ score. Dependent variable: SCQ mean score. R^2^ of the final model: 0.283, *p*-value < 0.001. Fixed variables in the model: AGU and EDMCQ. Variables excluded from the model by forward elimination (*p* ≥ 0.050): age (*p* = 0.798), gender (*p* = 0.234), work hours (*p* = 0.174), emotional exhaustion (*p* = 0.613), and professional role (*p* = 0.050). MODEL B with EDMCQ factor scores. Dependent variable: SCQ mean score. R^2^ of the final model: 0.279, *p*-value < 0.001. Fixed variable in the model: AGU. Variables excluded from the model by forward elimination (*p* ≥ 0.050): age (*p* = 0.614), gender (*p* = 0.334), work hours (*p* = 0.092), emotional exhaustion (*p* = 0.839), factor F1 “self-reflective and empowering leadership by the hospital” (*p* = 0.330), F3 “not avoiding end-of-life decisions” (*p* = 0.935), F5 “active involvement of nurses in end-of-life care and decision-making” (*p* = 0.117), F6 “active decision-making by team leaders” (*p* = 0.397), F7 “ethical awareness” (*p* = 0.640), and professional role (*p* = 0.054).

**Table 4:** Linear regression for Emotional Exhaustion in Acute Geriatric Units.

Variables	Unstandardized B	Coefficients Std. Error	Standardized Coefficients Beta	t	*p*-Value	Collinearity Tolerance	Statistics VIF
MODEL A with EDMCQ total score							
Acute Geriatric Unit	0.768	0.690	0.106	1.112	0.269	0.987	1.013
Ethical Climate (total EDMCQ)	−0.248	0.157	−0.152	−1.579	0.117	0.979	1.021
Professional role	−3.004	1.157	−0.251	−2.596	**0.011**	0.967	1.034
Constant	31.201	9.587		3.254	**0.002**		
MODEL B with the EDMCQ factor scores							
Acute Geriatric Unit	1.228	0.710	0.170	1.731	0.087	0.909	1.100
Professional role	−2.829	1.145	−0.236	−2.471	**0.015**	0.960	1.042
F4. Mutual respect within the interdisciplinary team	−2.180	0.946	−0.227	−2.304	**0.023**	0.905	1.104
Constant	30.590	6.706		4.561	**<0.001**		

Legend. Abbreviations: EDMCQ = Ethical Decision-Making Climate Questionnaire; AGU = Acute Geriatric Unit, SCQ = Sinclair Compassion Questionnaire; F = factor. Bold formatting is used to indicate significant values. MODEL A with total EDMCQ score. Dependent variable: Emotional exhaustion total score (higher score is more exhaustion). R^2^ of the final model: 0.115, *p*-value 0.007. Fixed variables in the model: AGU and EDMCQ total score. Variables excluded from the model by forward elimination (*p* ≥ 0.050): age (*p* = 0.252), gender (*p* = 0.988), work hours (*p* = 0.421), SCQ mean score (*p* = 0.294). MODEL B with EDMCQ factor scores. Dependent variable: Emotional exhaustion total score (higher score is more exhaustion). R^2^ of the final model: 0.139, *p*-value 0.002. Fixed variable in the model: AGU. Variables excluded from the model by forward elimination (*p* ≥ 0.050): age (*p* = 0.294), gender (*p* = 0.930), work hours (*p* = 0.364), SCQ mean score (*p* = 0.090), EDMCQ factor F1 “self-reflective and empowering leadership of doctors” (*p* = 0.833), F2 ‘Open interdisciplinary reflection’ (*p* = 0.128), F3 “not avoiding end-of-life decisions” (*p* = 0.200), F5 “active involvement of nurses in end-of-life care and decision-making” (*p* = 0.093), F6 “active decision-making by doctors” (*p* = 0.579) and F7 “ethical awareness” (*p* =0.136).

## Data Availability

The datasets used and/or analyzed during the current study are available from the corresponding author on reasonable request due to ethical reasons (more specifically, in the informed consent of the participants, it was stated that data would not be shared unless new ethical approval is given, and we promised the units that data would not be shared in order that the individual units could not be identified).

## Abbreviations

The following abbreviations are used in this manuscript:

AGU	Acute Geriatric Units
ANOVA	Analysis of Variance
EDMCQ	Ethical Decision-Making Climate Questionnaire
EE	Emotional Exhaustion
HCP	Healthcare Provider
SCQ	Sinclair Compassion Questionnaire
SCQ-SF-HCPTA	Sinclair Compassion Questionnaire-Short Form-Healthcare Provider Team Assessment

## Appendix A. Ethical Climate Measured by Total Ethical Decision-Making Climate Questionnaire (EDMCQ) Score

**Table A1:** Descriptive statistics of the EDMCQ score.

EDMCQ (30 Items) [Theoretical Range: 30–140]		Statistic	Std. Error
	Mean	59.38	0.62
	95% CI Lower Bound	58.16	
	95% CI Upper Bound	60.61	
	5% Trimmed Mean	59.50	
	Median	59.97	
	Variance	41.08	
	Std. Deviation	6.41	
	Minimum	40.49	
	Maximum	79.61	
	Range	39.12	
	Interquartile Range	7.74	
	Skewness	−0.29	0.23
	Kurtosis	0.87	0.46

**Table A2:** Agreement on the individual items of the EDMCQ (Factors 1, 3, 5, 6, 7).

	Total Sample (n = 108)	AGU a (n = 24)	AGU b (n = 25)	AGU c (n = 14)	AGU d (n = 21)	AGU e (n = 24)	*p*-Value
*Factor 1: Self-reflective and empowering leadership of team leaders* (mean score: 16.44, observed range: [10.02–22.15])							
In my unit, team leaders help team members settle their differences.	60 (55.6%)	16 (66.6%)	16 (64%)	9 (64.3%)	11 (52.4%)	8 (33.3%)	0.15
In my unit, team leaders trust the team members to exercise good judgement.	95 (88%)	23 (95.8%)	22 (88%)	13 (92.9%)	19 (90.5%)	18 (75%)	0.753
In my unit, team leaders permit the team members to use their own judgement in solving problems.	74 (68.5%)	18 (75%)	14 (56%)	9 (64.3%)	19 (90.5%)	14 (58.3%)	0.157
In my unit, team leaders encourage initiative in the team members.	82 (75.9%)	21 (87.5%)	20 (80%)	11 (78.5%)	17 (80.9%)	13 (54.2%)	0.362
In my unit, team leaders treat all team members as their equals.	73 (67.5%)	18 (75%)	16 (64%)	12 (85.7%)	16 (76.2%)	11 (45.8%)	0.243
In my unit, team leaders are well aware of their own emotions and attitudes.	55 (50.9%)	17 (70.9%)	13 (52%)	9 (64.3%)	11 (52.4%)	5 (20.8%)	0.089
In my unit, team leaders dare to show their vulnerability.	63 (58.3%)	13 (54.1%)	17 (68%)	9 (64.3%)	11 (52.4%)	13 (54.2%)	0.736
In my unit, team leaders are well aware of their role model function.	72 (66.7%)	19 (79.2%)	16 (64%)	11 (78.5%)	13 (61.9%)	13 (54.2%)	0.644
*Factor 3: Culture of not avoiding end-of-life decisions*							
(mean score: 3.83, observed range: [1.29–5.16])							
In my unit, death is not perceived as a treatment failure, so decisions to withdraw or withhold therapy are seldom postponed.	93 (86.1%)	21 (88.5%)	23 (92%)	13 (92.9%)	19 (90.5%)	17 (70.8%)	0.167
In my unit, EOL decisions are not frequently postponed.	81 (75%)	18 (75%)	18 (72%)	12 (85.7%)	17 (81%)	16 (66.7%)	0.248
*Factor 5: Active involvement of nurses in EOL care and decision-making* (mean score: 5.78, observed range: [3.67–8.20])							
In my unit, nurses are present during the communication of end-of-life information to the family.	70 (64.9%)	15 (62.5%)	17 (68%)	10 (71.4%)	15 (71.5%)	13 (54.1%)	0.887
In my unit, nurses are involved in end-of-life decision-making.	80 (74.1%)	18 (75%)	15 (60%)	10 (71.4%)	19 (90.5%)	18 (75%)	0.482
In my unit, nurses and physicians collaborate well with one another during end-of-life situations.	90 (83.4%)	17 (70.8%)	21 (84%)	10 (71.4%)	20 (95.2%)	22 (91.7%)	0.148
*Factor 6: Active decision-making by team leaders* (mean score: 5.85, observed range: [3.26–8.15])	61 (56.5%)	17 (70.8%)	10 (40%)	11 (78.5%)	14 (66.7%)	9 (37.5%)	0.212
In my unit, team leaders make accurate and timely decisions.							
In my unit, team leaders take full charge when emergencies arise.	67 (62%)	20 (83.3%)	11 (44%)	9 (64.2%)	16 (76.2%)	11 (45.8%)	**0.048**
In my unit, team leaders in charge are not hesitant about taking initiative in the group.	74 (68.6%)	19 (79.2%)	14 (56%)	13 (92.8%)	16 (76.2%)	12 (50%)	0.358
*Factor 7: Practice and culture of ethical awareness* (mean score: 5.21, observed range: [4.44–6.68])							
In my unit, my colleagues understand my thoughts/feelings about difficult end-of-life decisions.	97 (99.1%)	24 (100%)	25 (100%)	14 (100%)	21 (100%)	23 (95.8%)	0.6657
In my unit, different opinions and values concerning end of life are tolerated.	106 (98.1%)	24 (100%)	24 (96%)	13 (92.9%)	21 (100%)	24 (100%)	0.548
In my unit, we talk about moral problems.	98 (90.8%)	20 (83.3%)	23 (92%)	12 (85.7%)	21 (100%)	22 (91.7%)	0.644

Legend. AGU a–e = refers to the different participating AGU sites (intentionally anonymized); EDMCQ = Ethical Decision-Making Climate Questionnaire, 30-item questionnaire with theoretical range between 30 and 142. Bold formatting is used to indicate significant values.

## Appendix B. Team Performance in Compassionate Care Measured by Sinclair Compassion Questionnaire (SCQ) Score

**Table A3:** Descriptive statistics of the SCQ score.

SCQ (5 Items) [Theoretical Range: 1–5]		Statistic	Std. Error
	Mean	3.99	0.05
	95% CI Lower Bound	3.89	
	95% CI Upper Bound	4.08	
	5% Trimmed Mean	3.99	
	Median	4.00	
	Variance	0.24	
	Std. Deviation	0.49	
	Minimum	2.40	
	Maximum	5.00	
	Range	2.60	
	Interquartile Range	0.60	
	Skewness	−0.21	0.24
	Kurtosis	0.40	0.47

## Appendix C. Healthcare Providers’ Emotional Exhaustion (EE) Score

**Table A4:** Descriptive statistics of the EE score.

EE (9 Items) [Theoretical Range: 0–54]		Statistic	Std. Error
	Mean	13.91	0.95
	95% CI Lower Bound	12.03	
	95% CI Upper Bound	15.78	
	5% Trimmed Mean	13.12	
	Median	11	
	Variance	105.85	
	Std. Deviation	10.29	
	Minimum	0	
	Maximum	53	
	Range	53	
	Interquartile Range	14	
	Skewness	1.16	0.22
	Kurtosis	1.42	0.44
